# The Use of Chromagen Lenses in Different Ocular and Non-ocular Conditions: A Prospective Cohort Study

**DOI:** 10.7759/cureus.28963

**Published:** 2022-09-09

**Authors:** Zainab Alkhudairy, Fatemah Al Shamlan

**Affiliations:** 1 Orthoptics, Imam Abdulrahman Bin Faisal University Hospital, Dammam, SAU; 2 Ophthalmology, Dhahran Eye Specialist Hospital, Dhahran, SAU

**Keywords:** colored filters, chromagen lenses, irlen syndrome, dyslexia, cone-rod dystrophy, color vision deficiency

## Abstract

Background

In this study, we aimed to evaluate the efficacy of chromagen lenses and compare the pre- and post-intervention outcomes among individuals with non-ocular conditions such as dyslexia and Irlen syndrome and ocular conditions such as color vision deficiency (CVD) and cone-rod dystrophy (CRD).

Methodology

This prospective cohort study was conducted from 2016 to 2021 among cases (seven years or older) who were diagnosed with dyslexia, Irlen syndrome, CVD, or CRD. Participants were given a short questionnaire to read, followed by asking direct questions regarding medical health history, ocular history, eyeglasses prescription, and a full orthoptics evaluation. The main outcomes were the improvement in reading speed, reading accuracy, and visual stress.

Results

A total of 156 patients were included in this study; 110 patients with dyslexia, 19 with Irlen syndrome, 16 with CVD, and 11 with CRD. The findings showed that the reading speed and accuracy were improved in 96.34% of patients with dyslexia and 78.9% of patients with Irlen syndrome. The use of a chromagen lens was significantly associated with visual stress improvement in 89.8% of patients (p = 0.02). Photosensitivity was significantly improved after wearing the chromagen lenses in patients with CVD (87.5%) and CRD (63.6%).

Conclusions

The study findings showed a positive impact of chromagen lenses on reducing visual stress, including reading speed and accuracy, in patients with dyslexia and Irlen syndrome. Photosensitivity improved in patients with Irlen syndrome and CRD. Color vision was enhanced in patients with CVD. However, further studies are required to investigate the predictors of improvement and assess the long-term efficacy of chromagen lenses on daily activities and learning skills.

## Introduction

Dyslexia is a neurobiological condition characterized by difficulties with word recognition, poor spelling, and decoding abilities [1-3]. It is a reading deficiency impacting reading speed, comprehension, and reading eye movements in individuals with normal intelligence quotient (IQ), and it is three times more frequent in males compared to females [4,5]. When reading, the main complaints of visual stress include headaches, tiredness, difficulty coping with the blackboard at school, and slow reading [6].

Meares-Irlen syndrome is a visual perceptual dysfunction impacted by light, luminance, color contrast, and wavelength [7]. It is characterized by poor reading abilities and ineffective reading with limited comprehension [8]. Even though the symptoms are clearly described as visual stress symptoms (headache, photophobia, eye strain) and visual perceptual disorder (illusions of pattern, shapes, and colors), the precise nature of this syndrome has not been identified in the literature [9]. For the perception of color to occur, there has to be a harmonic relationship between the eyes and the brain [10,11]. The normal color vision is trichromatic because the eye has cones that are divided into three basic color pigments (red, green, and blue) [12].

Color vision deficiency (CVD) is a non-progressive, hereditary X-linked condition affecting 8% of males and 0.5% of females [13]. In affected individuals, the peak sensitivity of one of the red or green-sensitive cones is shifted to a different peak wavelength, or, in more severe cases, one photopigment is absent, producing more severe deficiency. Hereditary blue cone deficiency is rare but not uncommonly acquired in various diseases [14]. Cone-rod dystrophy (CRD) is an inherited retinal disorder that belongs to a group of pigmentary retinopathies [15], which is associated with many predominant symptoms such as decreased visual acuity, decreased sensitivity in the central visual field, photosensitivity, and CVD [16].

The four conditions (CRD, CVD, Irlen syndrome, and dyslexia) interfere with patients’ daily life, making their normal daily activities challenging. Therefore, many researchers proposed the use of colored lenses to reduce the associated symptoms and improve the quality of life. The concept of using colored lenses to improve vision outcomes is not new [17]. In the early 70s, the X-chrome lens was released. It is a soft-red-colored contact lens made from polymethyl methacrylate, which is worn on the non-dominant eye to create a brightness difference [18-20]. In addition, a study reported that blue-colored lenses had a significant effect on increasing the speed of reading in children with dyslexia [21]. Color lenses can improve the autonomic nervous system and treat the symptoms of poor reading [1]. In 1958, a study demonstrated significant improvement in the reading speed and accuracy of a student who could not detect words on a white paper when it was replaced with a yellow page [22]. Throughout the years, there has been a debate on the effectiveness of using colored lenses on patients with reading difficulties; however, it is commonly agreed that blue-colored lenses significantly increased the speed of reading in children with dyslexia [23,24]. Therefore, lenses and colored filters have been proposed as treatments to reduce the effect of bright contrast and improve patients’ reading skills [25]. This study aimed to evaluate the efficacy of chromagen lenses and compare the pre- and post-intervention outcomes among individuals with non-ocular conditions such as dyslexia and Irlen syndrome and ocular conditions such as CVD and CRD.

## Materials and methods

Study design

A prospective cohort study was conducted over five years from 2016 to 2021. Ethical approval to conduct the study was obtained from the Institutional Research Board (Bioethical Research Committee). In addition, written and verbal consent was obtained by all included patients or their parents.

The participants were recruited from awareness campaigns, school visits, or referrals from an ophthalmology clinic and tertiary hospitals from different parts of the country. Instructions on the color selection of the lenses were described in this study according to Harris’s guidance [26]. The color of lens selection was based on the patient’s subjective response in each case, and each lens was put on the non-dominant eye first. The subjective response was recorded according to the patient’s complaints and responses to the test before and after using lenses. For patients with color blindness and CRD, the lens was given to all patients to wear for a short time (20 minutes) to modify the tint color if needed.

Inclusion and exclusion criteria

In this study, patients aged seven years and older who were diagnosed with dyslexia, Irlen syndrome, CRD, or CVD were included. The age threshold of seven years was selected because at this age the reading progress of each participant can be predicted, the Wilkins reading test and Ishihara test can be performed and tested easily, and they can express symptoms verbally. Patients with the corrected refractive error were included to evaluate the best visual acuity near to be able to see and read the Wilkins reading test and the Ishihara numbers. In addition, the study included patients with good and poor vision. Any type of CRD, whose main symptom is photophobia, was included in the study. Participants were excluded if they had any mental disabilities, including autism or delayed speech.

Data collection

Participants were given a questionnaire in the Arabic language, which was adapted from the Australian Association of Irlen Institute (https://aaic.org.au/), followed by direct questioning regarding medical history, ocular history, and eyeglasses prescriptions. All participants were given varied colored lenses to determine the optimal color match that helps improve glare and photosensitivity; however, patients were aware that neither vision nor color vision would not improve with these lenses as these patients’ vision had been reduced due to the retinal disease in the case of CRD, for example. Then, a complete orthoptic evaluation was performed for each participant, including visual acuity, cover test, dry and wet refraction, and convergence near-point test.

For dyslexia, an IQ test was performed on each patient by other professionals outside the hospital to ensure that each participant was diagnosed with visual learning difficulties only, followed by the Wilkens rate of reading test, which is a conventional reading test for educational purposes only [27]. According to the Wilkens theory, the test was designed to be visually stressful while minimizing the linguistic and semantic aspects of reading. The text was printed in Times New Roman with a font size of nine and single-spaced, with a four-point (0.36 mm) horizontal spacing between words using Microsoft Word 5.0® on an Apple Machintosh® computer. The text was set as a paragraph 72.5 mm wide and 33.4 mm high, with an interline space of 3.15 mm. The letters had an x-height of 1.6 mm and a width average of 1.53 mm. About 15 common words were used in each line in a different random order, and it took less than two minutes to be read [28]. All the words used in the test were chosen from children’s reading books. The orthoptist recorded the reading speed, accuracy, and the number of errors, and the level of visual stress was assessed verbally by the patient before and after testing with chromagen lenses, which means that the reader would mention whether the symptoms of visual stress such as distortion of letters and loss of place when reading disappeared when wearing chrome lenses during the test.

Chromagen lenses and the reading test (Wilkins) were used to check speed, accuracy, and comfort level while reading through a standard black-and-white background. While changing the lens colors, the score of mistakes along with speed and accuracy was recorded.

For Irlen syndrome, the patients were referred by a neuro-ophthalmology clinic. Patients underwent a full medical history examination and were given a questionnaire to assess their visual stress symptoms. Each participant was asked if any color would help relax the eye and if it helped keep the person calm and relaxed physically around the fluorescent light situation in the clinic and while waiting in the hospital waiting area for 20 minutes. Moreover, the questionnaire was administered again to compare the results before and after wearing chromagen lenses.

For CVD, the patients were referred from other ophthalmology clinics after confirming their diagnosis. The participants were asked to read the Ishihara 24 plates with and without the chromagen lenses and score the mistakes and whether they could read the numbers correctly before and after wearing the lenses.

For CRD, the patients were diagnosed by retina specialists and then referred to our clinic with a report confirming their diagnosis of CRD in general without any specification of the type. Electroretinography (ERG) was performed for all patients, and the report was attached to their referral letter; however, the classification of the type of CRD was not mentioned. The referral aimed to prescribe colored lenses to eliminate severe photosensitivity caused by this retinal disorder. In our clinic, the participants were evaluated in a bright light environment (for example, in the clinic, and under the sun) for 20 minutes to assess the level of photophobia before and after trying the lenses and score the results using a 0-10 level sheet.

Statistical analysis

The sample size was calculated according to the total number of patients who presented with such symptoms to the orthoptic clinic. Using Cochran’s equation, a random sample of 139 patients in the target population was required. The normality of the collected data was examined using the Kolmogorov-Smirnov test and the Shapiro-Wilk test. Continuous variables were reported as mean and standard deviation (SD). Categorical data were reported as frequency and percentage. Comparisons between continuous variables were conducted using the Wilcoxon signed-rank test. The chi-square test and Fisher exact test were used to compare categorical variables. A p-value of <0.05 was considered statistically significant.

## Results

Demographic and clinical characteristics

This study included 156 participants; 110 participants with dyslexia, 19 with Irlen syndrome, 16 with CVD, and 11 with CRD. Table 1 shows the demographic and clinical characteristics of included participants.

**Table 1:** Demographic and clinical characteristics of participants. CRD: cone-rod dystrophy; CVD: color vision deficiency

Variables		Dyslexia	Irlen syndrome	CVD	CRD
Age (years)	Mean ± SD	12.15 ± 6.3	23.95 ± 11.6	30.25 ± 10.2	15.6 ± 9.8
	Minimum, maximum	7, 49	4, 39	14, 55	7, 35
Gender	Male	62 (56.4%)	8 (42.1%)	14 (87.5%)	3 (27.3%)
	Female	48 (43.6%)	11 (57.9%)	2 (12.5%)	8 (72.7%)
Color of lenses trial in clinic	Blue	45 (40.9%)	7 (36.8%)	-	-
	Aqua	14 (12.7%)	8 (42.1%)	-	-
	Green	1 (0.91%)	1 (5.26%)	-	-
	Yellow	22 (20%)	2 (10.5%)	-	-
	Violet	-	1 (5.26%)	-	-
	Magenta	-	-	15 (93.75%)	10 (90.9%)
	Orange	-	-	-	1 (9.09%)
	Pink	-	-	1 (6.25%)	-
	No lenses used	28 (25.5%)	-	-	-
	Total	110 (100%)	19 (100%)	16 (100%)	11 (100%)
Color of lenses visual stress improvement	Blue	43 (52.43%)	6 (31.6%)	-	-
	Aqua	14 (17.07%)	6 (31.6%)	-	-
	Yellow	21 (25.60%)	2 (10.5%)	-	-
	Magenta	-	-	13 (81.3%)	6 (54.5%)
	Green	1 (1.21%)	1 (5.26%)	-	-
	Orange	-	-	-	1 (9.09%)
	Violet	-	0 (0%)	-	-
	Pink	-	-	1 (6.25%)	-
	Total	79 (96.34%)	15 (78.9%)	14 (87.5%)	7 (63.6%)

Visual stress improvements in reading speed, accuracy, and photosensitivity

Visual stress was significantly (p < 0.05) improved in 96.34% of the patients with dyslexia and 78.9% of patients with Irlen syndrome when using the lenses. In CVD and CRD patients, the visual stress was improved in 87.5% and 63.6% of the patients, respectively, when using the lenses (Table 1).

Association between demographics and visual stress improvement

There was no significant difference between patients who improved and those who did not show significant improvement regarding age and gender (p = 0.693 and 0.428, respectively) (Table 2).

**Table 2:** Visual stress improvement in relation to age and gender in all patients. Fisher’s exact test and independent-sample t-test were used in this comparison.

Parameters	Variables	Improved	Not improved	P-value
Age	Mean ± SD	16.77 ± 10.71	18.00 ± 9.48	0.693
Gender	Male	61 (91.0%)	6 (9.0%)	0.428
	Female	54 (88.5%)	7 (11.5%)	

Association between using color lenses and visual stress

The analysis demonstrated that the majority of patients who used colored lenses showed improvement in visual stress (89.8% vs. 10.2%, p = 0.02) (Table 3).

**Table 3:** The relationship between using color lenses and visual stress improvement in all patients.

Lens color	Visual stress improved, N (%)	Visual stress not improved, N (%)	P-value
Total lenses	115 (89.8%)	13 (10.2%)	0.02
Blue	49 (94.2%)	3 (5.8%)	
Yellow	23 (95.8%)	1 (4.2%)	
Aqua	20 (90.9%)	2 (9.1%)	
Magenta	19 (76.0%)	6 (24.0%)	
Green	2 (100%)	0 (0%)	
Orange	1 (100%)	0 (0%)	
Violet	0 (0%)	1 (100%)	
Pink	1 (100%)	0 (0%)	

Ishihara bookplates test

**Figure 1:**
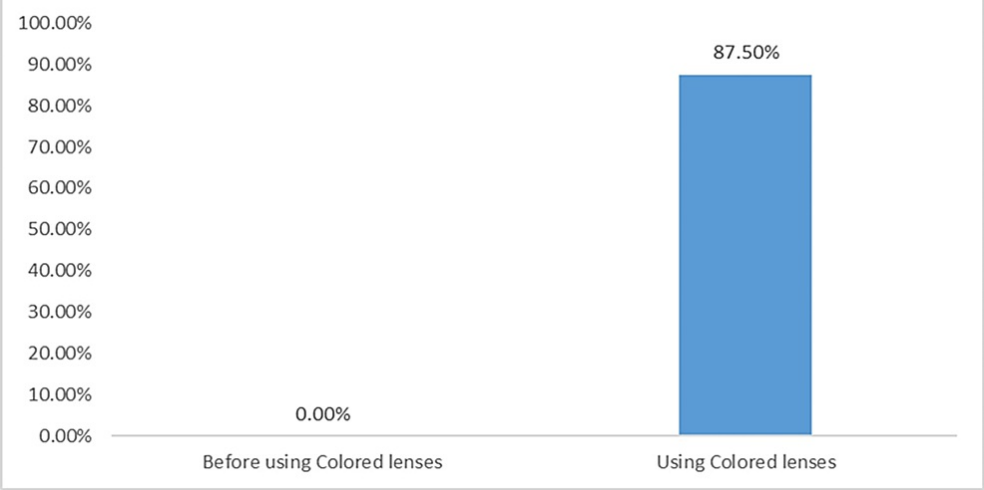
Ishihara color vision test before and after using the lens.

In patients with CVD, positive ranks showed that Ishihara plates were significantly improved in 14 (87.5%) patients after using the lenses, whereas only two patients showed no improvement before and after using the lenses (p < 0.001) (Table 4, Figure 1).

**Table 4:** Comparing color vision test (Ishihara bookplates) before and after using lenses in CVD patients. *: Wilcoxon singed-rank test. CVD: color vision deficiency

Rank test*	Before using lenses	After using lenses
Negative ranks	0	0
Positive ranks	0	14
Ties	2	2
Z-value	-3.341	
P-value	0.001	

In patients with CRD, we compared black sunglasses and chromagen lenses regarding photosensitivity (Table 5).

**Table 5:** Chi-square test for comparison between black sunglasses and chromogen lenses. OR: odds ratio

Glasses	No improvement, N (%)	Improved, N (%)
Black sunglasses	11 (100%)	0 (0%)
Chromagen lenses	4 (36.4%)	7 (63.6%)
Total	15 (68.1%)	7 (31.9%)
OR	2.75	
P-value	0.001	

The findings showed that chromagen lenses significantly improved the symptoms of seven (63.6%) cases versus no improvements in patients who used black sunglasses.

## Discussion

Educational activities mostly include reading and writing; therefore, dyslexia substantially influences children’s academic progress [29]. Children with dyslexia benefit from the usage of colored filters to minimize and prevent visual stress [28,30]. Therefore, in some cases of learning disabilities, colored filters may be described as assistive technology [31]. An example of colored filters is chromagen lenses which have been described as life-changing tools for people suffering from dyslexia, as well as an optical correction treatment for some cases of color deficiency, also known as color blindness [32]. The chromagen lenses are available in eight tints (blue, aqua, green, yellow, magenta, pink, orange, and violet) with different frequencies. The tints are available in light, medium, and dark densities [33]. They can be in the form of glasses, contact lenses, and clip-ons over glasses. They have been described as the most convenient and comfortable lenses to use because they are designed to cover the pupil area, making them cosmetically acceptable [32]. A study reported a marked improvement in symptoms and visual function using red contact lenses in 23 patients with cone disorders, especially symptoms related to photosensitivity [34]. However, there is no sufficient evidence regarding using chromagen lenses in CRD patients.

In a double-masked, randomized controlled trial, Harris and MacRow-Hill showed that using chromagen lenses was associated with a significant increase in the reading rate by decreasing the distortion of the text. These findings suggest that using colored filters in patients with dyslexia can improve reading speed and accuracy [35]. This study showed that the reading speed and accuracy were improved in 52.43% of the patients with dyslexia using blue-colored lenses. These findings are consistent with those of Chase et al., who reported that blue-colored lenses had a significant effect on increasing reading speed in children with dyslexia [36]. Ray et al. demonstrated that reading was markedly improved after three months of using a yellow filter in children with reading difficulties compared to the use of no filter. They considered colored filters to be an efficient intervention for delayed readers, suggesting that the yellow color stimulated the L- and M-cones within the magnocellular system [37]. Similarly, this study showed that 25.60% of the patients with dyslexia improved when using yellow filters. Likewise, our findings support the observation of Wilkins, who showed that colored plastic overlays and glasses could help eliminate visual stress, improve physical symptoms, and increase reading speed and accuracy [6].

Perceptual distortions, excessive brightness, and eyestrain headaches are the main symptoms of visual stress. It has been suggested that black text on a white background is associated with distortions and apparent text motion in patients with abnormal visual cortices [6]. Irlen stated that colored filters often change lives by improving reading abilities and enhancing academic performance [38].

On the other hand, there are limited studies regarding the impact of colored lenses and filters on photosensitivity in patients with Irlen syndrome. Most published studies have focused on their role in reading difficulties. Ritchie et al. demonstrated that Irlen colored overlays have no immediate positive impact on the reading rate test or the global reading measure [39]. This study focused on the role of using chromagen lenses in improving visual stress symptoms, which showed significant effects among 78.9% of Irlen syndrome patients after using colored lenses.

Regarding patients with CVD, this study showed that 87.5% of the patients had significantly (p < 0.001) improved in reading the Ishihara color vision test after using chromagen lenses. Oriowo and Alotaibi investigated the role of chromagen spectacle lenses in patients with CVD using the Ishihara color test. Their findings showed that chromagen lenses can improve color vision perception in some cases of CVDs [32]. In their study, improvement was observed in 84.61% of patients, which supports the finding of this study. Additionally, a study by Hodd et al. showed that chromagen lenses could improve color perception in some individuals by making colors appear brighter and more obvious [40].

This study demonstrated that all patients with CRD showed improvement in visual stress and photosensitivity when they used the magenta-colored filters (90.91%) and orange-colored filters (9.09%). A study investigated the effectiveness of red lenses and gray lenses in reducing photosensitivity in two patients with CRD. According to the study, red filters can improve both visual acuity and field [36]. Another study reported a marked improvement in symptoms and ineffective visual function using red contact lenses in 23 patients with cone disorders. The study reported that red contact lenses immediately resolved aversion to light, with dramatically improved visual function in all patients. Furthermore, 35% of the included patients were able to apply for a driver’s license after using colored filters [34]. Severinsky et al. evaluated the impact of centrally red-tinted hydrogel contact lenses on nine patients with severe photophobia and poor visual acuity. They reported that all patients described a major improvement in their photophobia both outdoors and indoors, as well as a marked improvement in quality of life. They recommended using these colored filters as a part of the regular assessment in specialty clinics treating patients with low vision, glare, and photophobia [41]. A case report of a 28-year-old patient with CRD showed that a red contact lens was associated with substantial improvement in visual performance, photophobia, and contrast sensitivity [42].

We acknowledge that this study has some limitations, including the small sample size in each subgroup and the single-center setting, which may hinder the generalizability of our findings.

## Conclusions

This study highlights the positive impact of using colored lenses on reading speed, accuracy, and photosensitivity in patients with dyslexia, Irlen syndrome, CVD, and CRD. The use of a chromagen lens was significantly associated with visual stress improvement. Photosensitivity significantly improved after wearing the chromagen lenses in patients with CVD and CRD. However, further studies are required to investigate the predictors of improvement and assess the long-term efficacy of chromagen lenses on daily activity and learning skills.
